# Benefit of splenectomy in distal pancreatectomy for neuroendocrine tumours: multicentre retrospective study

**DOI:** 10.1093/bjsopen/zraf038

**Published:** 2025-05-13

**Authors:** Elise Clément, Pietro Addeo, Alain Sauvanet, Célia Turco, Ugo Marchese, Safi Dokmak, Christophe Laurent, Ahmet Ayav, Olivier Turrini, Laurent Sulpice, Régis Souche, Julie Perinel, David J Birnbaum, Olivier Facy, Johan Gagnière, Lilian Schwarz, Guillaume Piessen, Nicolas Regenet, Antonio Iannelli, Jean Marc Regimbeau, Xavier Lenne, Bruno Heyd, Sébastien Gaujoux, Mehdi El Amrani, Alexandre Doussot, Collaborators, Mustapha Adham, Marie André, Philippe Bachellier, Louise Barbier, Thomas Bardol, Zineb Cherkaoui, Thibault Durin, David Fuks, Zaher Lakkis, Cloé Magallon, Fabien Robin, Edouard Roussel, Ecoline Tribillon, Stéphanie Truant, Stylianos Tzedakis

**Affiliations:** Department of Digestive Surgical Oncology, Liver Transplantation Unit, CHU Besançon, Besançon, France; Hepato-Pancreato-Biliary Surgery and Liver Transplantation, Pôle des Pathologies Digestives, Hépatiques et de la Transplantation, Hôpital de Hautepierre-Hôpitaux Universitaires de Strasbourg, Université de Strasbourg, Strasbourg, France; Department of HPB Surgery, Hôpital Beaujon, University of Paris, Clichy, France; Department of Digestive Surgical Oncology, Liver Transplantation Unit, CHU Besançon, Besançon, France; Department of Digestive, Hepatobiliary and Pancreatic Surgery, Cochin Teaching Hospital, AP-HP, Paris, France; Department of HPB Surgery, Hôpital Beaujon, University of Paris, Clichy, France; Department of Digestive Surgery, Hepatobiliary and Pancreatic Surgery Unit. Centre Magellan—CHU Bordeaux, Bordeaux, France; Department of HPB Surgery, Nancy University Hospital, Nancy, France; Department of Oncological Surgery, Institut Paoli Calmettes, Marseille University, Marseille, France; Department of Hepatobiliary and Digestive Surgery, University Hospital, Rennes 1 University, Rennes, France; Department of Surgery, Hopital Saint Eloi, Montpellier, France; Department of Digestive Surgery, Hopital Edouard Herriot, Lyon, France; Department of Digestive Surgery, Hôpital Nord, Assistance Publique-Hôpitaux de Marseille, Aix-Marseille University, Marseille, France; Department of Digestive and Surgical Oncology, University Hospital, Dijon, France; Department of Digestive and Hepatobiliary Surgery—Liver transplantation, University Hospital Clermont-Ferrand, Clermont-Ferrand, France; Department of Digestive Surgery, Rouen University Hospital and Université de Rouen Normandie, Rouen, France; Department of Digestive and Oncological Surgery, CHU Lille, Claude Huriez University Hospital, Lille, France; Department of Digestive Surgery, Nantes Hospital, Nantes, France; Digestive Surgery and Liver Transplantation Unit, University Hospital of Nice, Nice, France; Department of Digestive Surgery, Amiens University Medical Centre and Jules Verne University of Picardie, Amiens, France; Medical Information Department, Lille University Hospital, Lille, France; Department of Digestive Surgical Oncology, Liver Transplantation Unit, CHU Besançon, Besançon, France; Department of Hepato-Biliary and Pancreatic Surgery and Liver Transplantation, AP-HP, Pitié-Salpêtrière Hospital, Paris, France; Department of Digestive Surgery and Transplantation, Lille University Hospital, Lille, France; Department of Digestive Surgical Oncology, Liver Transplantation Unit, CHU Besançon, Besançon, France; Université de Franche-Comté, EFS, INSERM, UMR RIGHT, Besançon, France

## Abstract

**Background:**

Distal pancreatectomy is frequently indicated for left-sided pancreatic neuroendocrine tumour (NET). When combined lymphadenectomy is warranted, distal pancreatectomy with splenectomy (DPS) is generally advocated to optimize lymph node dissection. The spleen-preserving distal pancreatectomy (SPDP) may represent an alternative approach. This study aimed to evaluate postoperative and oncological results of distal pancreatectomy with and without splenectomy for pancreatic NET.

**Methods:**

This multicentre retrospective study included all distal pancreatectomy for pancreatic NET performed between 2014 and 2018. Patients with functional NET or multiple endocrine neoplasia type 1 were excluded. Indications and results were compared between DPS, distal pancreatectomy according to Kimura (K-SPDP) and distal pancreatectomy according to Warshaw (W-SPDP), before and after propensity score matching (PSM).

**Results:**

Among 251 patients included (108 DPS (43%), 73 K-SPDP (29%), and 70 W-SPDP (28%)), there was no difference in terms of patients’ characteristics, surgical approach, and conversion. Tumour size (*P* = 0.005), grade (*P* < 0.001) and the number of nodes analysed (*P* < 0.001) were significantly lower in patients undergoing K-SPDP as compared to W-SPDP or DPS. Apart from a difference in readmission rate (*P* = 0.002), there was no difference in terms of mortality rate or severe morbidity rate between the three techniques. After PSM comparing DPS (*n* = 70) and W-SPDP (*n* = 70), there was no difference in morbidity and mortality rates. R0 resection rate (91% *versus* 97%; *P* = 0.165), the number of nodes analysed (8 *versus* 7; *P* = 0.495), and median overall survival (*P* = 0.493) were not different.

**Conclusion:**

In cases of distal pancreatectomy for NET, splenectomy did not seem to improve lymph node dissection or survival. When lymph node dissection associated with distal pancreatectomy is justified, the benefit of splenectomy appears questionable.

## Teaser

When lymph node dissection associated with distal pancreatectomy for left-sided pancreatic neuroendocrine tumour is justified, the benefit of splenectomy appears questionable. Indeed, as compared to spleen-preserving vessel-resecting distal pancreatectomy, distal pancreatosplenectomy does not confer any clear advantage in terms of safety, lymph node clearance or survival.

## Introduction

Pancreatic neuroendocrine tumour (PNET) prognosis remains acceptable with median overall survival classically exceeding 10 years. Still, the presence of nodal metastases is a well-established risk factor of recurrence and decreased survival^1^. In case of left-sided grade 1 or 2 PNET larger than 2 cm without distant lesion, a distal pancreatectomy (DP) with regional lymph nodes dissection (LND) is indicated. European guidelines from the European Neuroendocrine Tumour Society do not define the type of LND for PNET^2^. Conversely, North American Neuroendocrine Tumor Society guidelines are rather centred on the quality of the dissection with a recommended number of lymph nodes ranging between 11 and 15, without recommending clearly which technique should be preferred^3^. The splenic preservation during DP may be technically demanding, could be associated with post-pancreatectomy haemorrhage or spleen infarction, and may also limit nodal retrieval. The spleen preserving distal pancreatectomy (SPDP) may preserve patients’ innate immune responses and some have argued that it is associated eith fewer complications than distal splenopancreatectomy (DPS)^4–6^. Further, although the lymphatic drainage pathway of the pancreatic body and tail is mostly directed towards the coeliac trunk, there are no data warranting routine splenectomy for left-sided PNET resection.

The current study aimed to evaluate the impact of DP techniques for non-functional PNET in terms of short- and long-term outcomes and on LND.

## Methods

### Study population

This was a retrospective and multicentre study carried out in high-volume referral centres, defined as those performing more than 20 pancreatectomies per year^7^. Patients aged ≥18 years were included if they underwent elective distal pancreatectomy for PNET, between 2014 and 2018, with or without spleen preservation, with open or minimally invasive approach. Patients who underwent DP in the setting of multiple endocrine neoplasia type 1 (MEN1), functioning PNETs, and extended DP as defined by the International Study Group for Pancreatic Surgery (ISGPS) were excluded^8^. Different DP techniques were defined as DPS (harvesting all lymph nodes along splenic vessels including those at the splenic hilum), SPDP with splenic vessels preservation according to the Kimura technique (K-SPDP, harvesting lymph nodes along the splenic vessels while preserving those vessels), and SPDP with splenic vessels resection according to the Warshaw technique (W-SPDP, harvesting all lymph nodes with resected splenic vessels while leaving in place those at the splenic hilum)^9,10^.

### Data collection

Demographic and baseline characteristics regarding patients and tumour features were collected. Intraoperative variables (type of surgical approach, operative time, estimated blood loss, spleen preservation techniques), data on final pathology (tumour size, tumour grade, nodal status, and resection margin) were also retrieved. Complete resection (R0) margin was defined as surgical margin larger than 1 mm. Postoperative mortality and morbidity rates within 90 days were recorded according to the Clavien–Dindo classification with severe morbidity rate corresponding to morbidity grade greater than 2^11^. Pancreas-specific postoperative complications including postoperative pancreatic fistula (POPF), delayed gastric emptying, and post-pancreatectomy haemorrhage (PPH) were defined according to ISGPS definitions^12–14^. Similarly, clinically relevant splenic complications defined as splenic ischaemia or haemorrhage requiring reoperation for splenectomy were collected. In case of postoperative complication, reintervention was defined as either involving interventional radiology, endoscopy, or surgery. Reason for any reintervention was monitored. Oncological follow-up with data concerning tumour recurrence and overall survival were also collected.

### Management and outcomes

All patients were managed according to national guidelines and decision for DP was discussed at dedicated multidisciplinary tumour boards in each centre^15^. Preoperative work-up comprised systematically thoracoabdominal CT, liver magnetic resonance imaging, and ^68^Ga-DOTATATE PET/CT to detect regional and distant metastases. Decision for indication and techniques of spleen preservation was at the discretion of the primary surgeon at each centre.

### Statistics analysis

Categorical variables, presented as numbers and percentages, were compared using the χ^2^ test. Continuous variables with a normal distribution are presented as mean(s.d.) and non-normally distributed variables as median (i.q.r.), and were compared using the Kruskal–Wallis test. Comparisons between resection types were performed regarding preoperative and intraoperative data and postoperative outcomes in the full cohort. Additionally, due to obvious differences between patients who underwent K-SPDP and those in both the W-SPDP and DPS groups, patients in the W-SPDP group were compared to those undergoing DPS, before and after matching using the propensity score matching (PSM) method. The propensity score for an individual was calculated using a multivariable logistic regression model including the following variables: age, gender, body mass index, ASA score, tumour size, and tumour grade. Using a standard calliper width of 0.2, patients were matched without replacement to the closest matching propensity score in a 1 : 1 ratio.

Patients who died within 90 days after surgery were excluded from survival analysis. Overall survival (OS) was calculated from the date of resection to the date of death, or the date of last follow-up, and recurrence-free survival (RFS) was calculated from the date of resection to the date of first recurrence, the date of death, or last follow-up. OS and RFS were estimated using the Kaplan–Meier method and compared between groups using the Log-rank test. Two-tailed *P* < 0.05 were considered statistically significant. Statistical analyses were carried out using SPSS Statistics 27.0 (IBM). The present study complied with the RECORD guidelines^16^.

## Results

### Study population

Over the study period, 272 patients were operated for PNETs in 21 hospitals, of whom 16 patients with MEN1 and 5 patients with functioning PNETs were excluded. Of 251 patients included in the study, 108 (43%) underwent DPS, 73 (29.1%) K-SPDP, and 70 (27.9%) W-SPDP (*Fig. S1*). Patients’ characteristics, tumour features, and perioperative outcomes are listed in *Table 1*. Overall, 133 resections (53%) were performed minimally invasively including 110 laparoscopic resections and 23 robotic resections. Conversion rate was 9% (*n* = 12) and reasons for conversion to open surgery were exposure difficulty (*n* = 7) and intraoperative bleeding (*n* = 5). Following surgery, 90-day mortality and severe morbidity rates were 1.2% (*n* = 3) and 13.9% (*n* = 35) respectively. Clinically relevant POPF and PPH rates were 18.3% (*n* = 46) and 5.6% (*n* = 14) respectively and 31 patients (12.4%) required reintervention. One patient experienced clinically relevant splenic complication requiring rescue splenectomy 11 days after W-SPDP, in the setting of POPF combined with PPH and arterial embolization failure. Median length of hospital stay was 10 days (range 4–242). Readmission rate was 13.1% (*n* = 33). Regarding tumour features, median tumour size was 25 mm (i.q.r. 15–40). The majority of PNET (92.4%, *n* = 232) were G1 or G2. R0 resection rate was 94% (*n* = 236).

**Table 1:** Patient characteristics and perioperative outcomes in the whole cohort (*n* = 251)

	Overall (*n* = 251)	DPS (*n* = 108)	K-SPDP (*n* = 73)	W-SPDP (*n* = 70)	*P*
**Patient outcomes**					
Age (years), median (i.q.r.)	60 (50–67)	62 (52–68)	60 (52–67)	59 (47–66)	0.291
Gender, female	129 (51.4%)	50 (46.3%)	39 (53%)	40 (57%)	0.338
BMI (kg/m^2^), median (i.q.r.)	26 (23.2–29.7)	26 (23.2–29.7)	26.5 (23.5–29.7)	25.7 (21.5–30)	0.733
ASA score > 2	21 (8.4%)	12 (11.1%)	5 (7%)	4 (6%)	0.382
Diabetes mellitus	37 (14.7%)	15 (13.9%)	13 (18%)	9 (13%)	0.668
Cardiovascular disease	16 (6.4%)	6 (5.6%)	4 (5%)	6 (7%)	0.675
**Intraoperative outcomes**					
Minimally invasive	118 (47%)	55 (50.9%)	31 (42%)	38 (54%)	0.518
Pancreatic neck section	158 (62.9%)	81 (75%)	36 (49%)	41 (59%)	0.001
Operative time (min), median (i.q.r.)	180 (140–227)	183 (149–240)	164 (140–240)	173 (120–210)	0.080
Blood transfusion	9 (3.6%)	5 (4.6%)	2 (3%)	2 (3%)	0.741
Estimated blood loss (ml), median (i.q.r.)	200 (100–400)	200 (100–400)	200 (77–387)	150 (100–400)	0.460
**Postoperative outcomes**					
90-day mortality	3 (1.2%)	2 (1.9%)	0	1 (1%)	0.520
Reintervention	31 (12.4%)	12 (11.1%)	7 (10%)	12 (17%)	0.173
90-day Clavien–Dindo grade ≥3	35 (13.9%)	14 (13%)	8 (11%)	13 (19%)	0.391
Clinically relevant PPH	14 (5.6%)	6 (5.6%)	2 (3%)	6 (9%)	0.130
Clinically relevant POPF	46 (18.3%)	21 (19.4%)	12 (16%)	13 (19%)	0.875
Hospital stay (days), median (i.q.r.)	10 (8–15)	11 (8–17)	11 (8–15)	9 (7–13)	0.048
Hospital readmission rate	33 (13.1%)	14 (13%)	16 (22%)	3 (4%)	0.002
**Tumour outcomes**					
Tumour size (mm), median (i.q.r.)	25 (14–40)	30 (20–50)	15.5 (11–24)	25 (16–40)	<0.001
Grade					0.005
Grade 1	128 (51%)	40 (37%)	54 (74%)	34 (49%)	
Grade 2	104 (41.4%)	55 (50.9%)	16 (22%)	33 (47%)	
Grade 3	8 (3.2%)	7 (6.5%)	0	1 (1%)	
Unknown	11 (4.4%)	6 (5.6%)	3 (4%)	2 (3%)	
R0 resection	236 (94%)	98 (90.7%)	70 (96%)	68 (97%)	0.154
Number of retrieved lymph nodes, median (i.q.r.)	5 (0–41)	8 (3–13)	1 (0–7)	7 (1–14)	<0.001

DPS, distal pancreatectomy with splenectomy; K-SPDP, Kimura spleen-preserving distal pancreatectomy; W-SPDP, Warshaw spleen-preserving distal pancreatectomy; BMI, body mass index; ASA, American Society of Anesthesiologists; POPF, postoperative pancreatic fistula; PPH, postpancreatectomy haemorrhage.

### Differences according to resection types

**Fig. 1:**
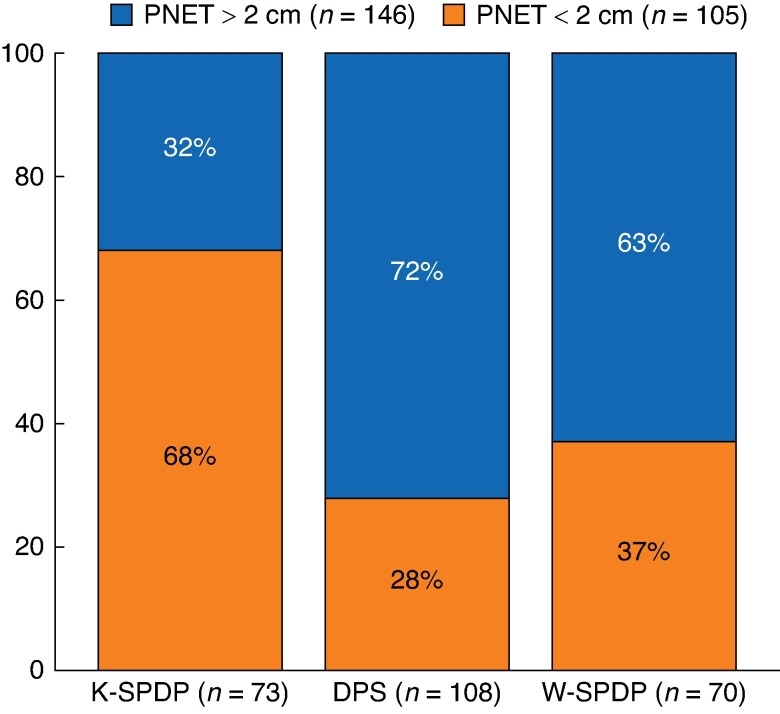
Distribution of tumour size according to the type of resection

PNET, pancreatic neuroendocrine tumour; K-SPDP, Kimura spleen-preserving distal pancreatectomy; DPS, distal pancreatectomy with splenectomy; W-SPDP, Warshaw spleen-preserving distal pancreatectomy.

There was no difference in terms of patients’ characteristics and surgical approach between the three types of resections. Intraoperatively, pancreatic transection at the neck was significantly more frequently performed in case of DPS (*P* < 0.001). Following surgery, there was no difference regarding mortality and severe morbidity rates, except in terms of median hospital stay (*P* = 0.048) and readmission rate (*P* = 0.002). Tumour features were significantly different according to the type of resection. Tumours were significantly smaller in patients who underwent K-SPDP (median: 15.5 mm, i.q.r. 11–24) as compared to those in the W-SPDP (median: 25 mm, i.q.r. 16–40) or DPS (30 mm, i.q.r. 20–50) groups respectively (*P* < 0.001). Considering a 2-cm size cut-off, K-SPDP was mostly performed for PNET <2 cm whereas both W-SPDP and DPS were performed for PNET >2 cm (*P* < 0.001; *Fig. 1*). Similar differences were observed regarding tumour grade, with 74% (*n* = 54) of G1 PNET in K-SPDP patients as compared to those in the W-SPDP (48.6%, *n* = 34) or DPS (37%, *n* = 40) groups respectively (*P* = 0.005).

With respect to lymph node dissection, at least one lymph node could be examined in 95 patients (88%) who underwent DPS, 58 patients (82.9%) who underwent W-SPDP, and 37 patients (50.7%) after K-SPDP (*P* < 0.001). When lymph node dissection was carried out (*n* = 190, 75.7%), there was a significant difference in the median number of harvested lymph nodes between DP types (*P* < 0.001). Among patients with at least one retrieved lymph nodes, 56 patients (29.5%) had nodal disease. Nodal disease was associated with tumour size (pN0 = 22 mm, i.q.r. 15–35; pN1 = 40 mm, i.q.r. 25–50; *P* < 0.001).

**Fig. 2:**
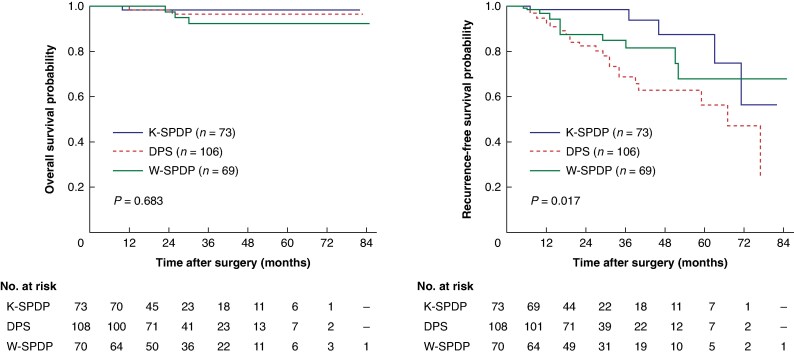
Overall and recurrence-free survival estimates in the whole cohort (*n* = 251)

K-SPDP, Kimura spleen-preserving distal pancreatectomy; DPS, distal pancreatectomy with splenectomy; W-SPDP, Warshaw spleen-preserving distal pancreatectomy.

Regarding long-term outcomes (*Fig. 2*), median follow-up was 24 months (i.q.r. 16–41). There was no difference in terms of OS (*P* = 0.683) whereas there was a statistically significant difference in median RFS estimates (DPS = 64 months, W-SPDP = not reached, K-SPDP = not reached; *P* = 0.017).

### Comparison between W-SPDP and DPS

**Fig. 3:**
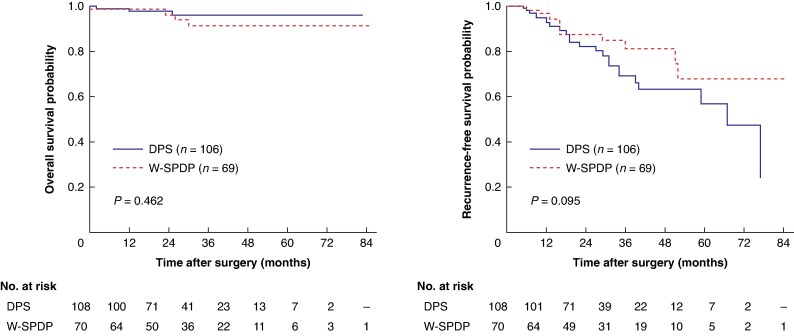
Overall and recurrence-free survival estimates in patients who underwent DPS or W-SPDP before propensity score matching

DPS, distal pancreatectomy with splenectomy; W-SPDP, Warshaw spleen-preserving distal pancreatectomy.

After excluding patients who underwent K-SPDP, patients who underwent W-SPDP (*n* = 70) were compared to those with DPS (*n* = 108) before PSM (*Table 2*). No statistically significant difference between the two groups was demonstrated except regarding operative time (*P* = 0.027) and length of hospital stay (*P* = 0.017) that were shorter in the W-SPDP group. There was no difference observed in terms of tumour size (*P* = 0.162) or tumour grade (*P* = 0.205). Similarly, there was no difference regarding R0 resection rate (*P* = 0.129) and the number of retrieved lymph nodes (*P* = 0.278). Regarding survival data, there was no difference in terms of OS (*P* = 0.462) and RFS (*P* = 0.095) between DP (*Fig. 3*).

**Table 2:** Comparison between distal pancreatosplenectomy and Warshaw technique before propensity score matching

	Overall (*n* = 178)	DPS (*n* = 108)	W-SPDP (*n* = 70)	*P*
**Patient outcomes**				
Age (years), median (i.q.r.)	61 (50–67)	62 (52–68)	59 (47–66)	0.131
Gender, female	90 (50.6%)	50 (46.3%)	40 (57%)	0.157
BMI (kg/m^2^), median (i.q.r.)	26 (22.9–29.7)	26 (23.2–29.7)	25.7 (21.5–30)	0.838
ASA score > 2	16 (9%)	12 (11.1%)	4 (6%)	0.219
Diabetes mellitus	24 (13.5%)	15 (13.9%)	9 (13%)	0.844
Cardiovascular disease	12 (6.7%)	6 (5.6%)	6 (7%)	0.433
**Intraoperative outcomes**				
Minimally invasive	87 (48.9%)	55 (50.9%)	38 (54%)	0.497
Pancreatic neck section	122 (68.5%)	81 (75%)	41 (59%)	0.021
Operative time (min), median (i.q.r.)	180 (140–222)	183 (149–240)	173 (120–210)	0.027
Blood transfusion	7 (3.9%)	5 (4.6%)	2 (3%)	0.552
Estimated blood loss (ml), median (i.q.r.)	200 (100–400)	200 (100–400)	150 (100–400)	0.251
**Postoperative outcomes**				
90-day mortality	3 (1.7%)	2 (1.9%)	1 (1%)	0.830
Reintervention	24 (13.5%)	12 (11.1%)	12 (17%)	0.250
90-day Clavien–Dindo grade ≥3	27 (15.2%)	14 (13%)	13 (19%)	0.308
Clinically relevant PPH	12 (6.7%)	6 (5.6%)	6 (9%)	0.433
Clinically relevant POPF	34 (19.1%)	21 (19.4%)	13 (19%)	0.885
Hospital stay (days), median (i.q.r.)	10 (8–15)	11 (8–17)	9 (7–13)	0.017
Hospital readmission rate	17 (9.6%)	14 (13%)	3 (4%)	0.054
**Tumour outcomes**				
Tumour size (mm), median (i.q.r.)	30 (20–45)	28 (20–50)	25 (16–40)	0.162
Grade				0.205
G1	74 (41.6%)	40 (37%)	34 (49%)	
G2	88 (49.4%)	55 (50.9%)	33 (47%)	
G3	8 (4.5%)	7 (6.5%)	1 (1%)	
Unknown	8 (4.5%)	6 (5.6%)	2 (3%)	
R0 resection	166 (93.3%)	98 (90.7%)	68 (97%)	0.129
Number of analysed lymph nodes, median (i.q.r.)	7 (2–14)	8 (3–13)	7 (1–14)	0.278
Lymph node status				0.065
pN0	96 (53.9%)	52 (48.1%)	44 (63%)	
pN1–2	53 (29.8%)	39 (36.1%)	14 (20%)	
pNx	29 (16.3%)	17 (15.7%)	12 (17%)	

DPS, distal pancreatectomy with splenectomy; W-SPDP, Warshaw spleen-preserving distal pancreatectomy; BMI, body mass index; ASA, American Society of Anesthesiologists; POPF, postoperative pancreatic fistula; PPH, postpancreatectomy haemorrhage.

**Fig. 4:**
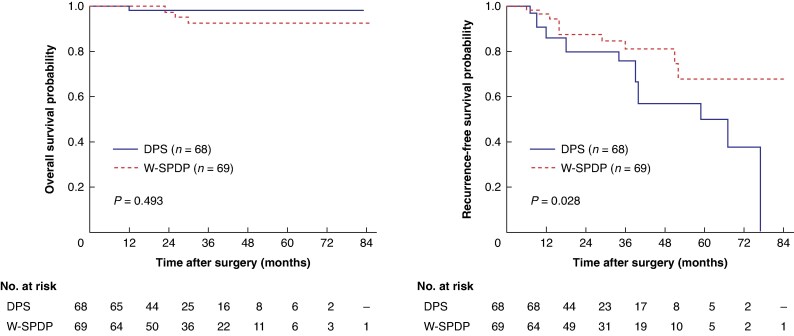
**Overall and recurrence-free survival estimates in patients who underwent DPS or W-SPDP after propensity score matching**DPS, distal pancreatectomy with splenectomy; W-SPDP, Warshaw spleen-preserving distal pancreatectomy.

After PSM, patients who underwent W-SPDP (*n* = 70) were compared to those with DPS (*n* = 70) (*Table 3*). Overall, there was no difference regarding all perioperative variables except regarding operative time (*P* = 0.011). Regarding survival data, there was no difference in terms of overall survival (*P* = 0.493), but median RFS was shorter in the DPS group (59 months) as compared to the W-SPDP group (not reached; *P* = 0.028) (*Fig. 4*).

**Table 3:** Comparison between distal pancreatosplenectomy and Warshaw technique after propensity score matching

	Overall (*n* = 140)	DPS (*n* = 70)	W-SPDP (*n* = 70)	*P*
**Patient outcomes**				
Age (years), median (i.q.r.)	60 (50–66)	61 (54–68)	59 (47–66)	0.201
Gender, female	77 (55%)	37 (53%)	40 (57%)	0.386
BMI (kg/m^2^), median (i.q.r.)	25.8 (25.7–30)	25.8 (23.2–29.8)	25.7 (21.5–30)	0.672
ASA score > 2	10 (7.1%)	6 (8%)	4 (6%)	0.327
Diabetes mellitus	21 (15%)	12 (17%)	9 (13%)	0.478
Cardiovascular disease	9 (6.4%)	3 (4%)	6 (9%)	0.301
**Intraoperative outcomes**				
Minimally invasive	76 (54.3%)	38 (54%)	38 (54%)	0.999
Transection at the neck	90 (64.3%)	49 (70%)	41 (59%)	0.158
Operative time (min), median (i.q.r.)	180 (179–240)	203 (148–250)	173 (120–210)	0.011
Blood transfusion	9 (6.4%)	7 (10%)	2 (3%)	0.085
Estimated blood loss (ml), median (i.q.r.)	200 (100–400)	200 (62–400)	150 (100–400)	0.436
**Postoperative outcomes**				
90-day mortality	3 (2.1%)	2 (3%)	1 (1%)	0.559
Reintervention	20 (14.2%)	8 (11%)	12 (17%)	0.179
90-day Clavien–Dindo grade ≥3	23 (16.4%)	10 (14%)	13 (19%)	0.278
Clinically relevant PPH	10 (7.1%)	4 (6%)	6 (9%)	0.275
Clinically relevant POPF	26 (18.6%)	13 (19%)	13 (19%)	0.999
Hospital stay (days), median (i.q.r.)	10 (7–15)	10 (8–16)	9 (7–13)	0.066
Hospital readmission rate	17 (12.1%)	14 (20%)	3 (4%)	0.004
**Tumour outcomes**				
Tumour size (mm), median (i.q.r.)	27 (16–40)	27 (25–45)	25 (16–40)	0.404
Grade				0.367
G1	65 (46.4%)	31 (44%)	34 (49%)	
G2	69 (49.3%)	36 (51%)	33 (47%)	
G3	2 (0.1%)	1 (1%)	1 (1%)	
Unknown	4 (0.3%)	2 (3%)	2 (3%)	
R0 resection	132 (94.3%)	64 (91%)	68 (97%)	0.145
Number of analysed lymph nodes, median (i.q.r.)	7 (2–14)	8 (2–13)	7 (1–14)	0.476
Lymph node status				0.168
pN0	85 (60.7%)	41 (59%)	44 (63%)	
pN1–2	33 (23.6%)	19 (27%)	14 (20%)	
pNx	22 (15.7%)	10 (14%)	12 (17%)	

DPS, distal pancreatectomy with splenectomy; W-SPDP, Warshaw spleen-preserving distal pancreatectomy; BMI, body mass index; ASA, American Society of Anesthesiologists; POPF, postoperative pancreatic fistula; PPH, postpancreatectomy haemorrhage.

## Discussion

In the current series, there was an obvious difference in the indications of distal pancreatectomy with or without spleen preservation techniques. K-SPDP was mostly performed in patients with smaller PNET, mostly G1, irrespective of patients’ characteristics. Pancreatic section was significantly more frequently performed at the neck in case of DPS, despite the absence of difference in terms of tumour location between groups. There was no difference in terms of mortality and severe morbidity rates between the three techniques and there was only a marginal difference in terms of length of hospital stay and readmission rate, favouring W-SPDP.

DPS is generally seen as the technique of choice for the sake of oncological radicality, especially regarding lymph nodes dissection. Regarding the PNET, Sahara and colleagues showed spleen preservation as associated with comparable or even better perioperative outcomes than DPS^17^, including patients operated at expert centres performing only W-SPDP. They concluded that W-SPDP might be better considered in selected cases, such as PNET <2 cm, centrally located in the pancreas. And SPDP should be avoided in tumours invading the splenic vessels or with suspicious lymph nodes in the splenic hilum. In the current study, there was no difference in terms of OS between W-SPDP and DPS. Nevertheless, there was a trend towards longer RFS in the W-SPDP group as compared to DPS before PSM, and this difference became statistically significant after PSM. Although long-term follow-up limited to a median time of 24 months should restrict any conclusion on long-term oncological outcomes, one could hypothesize that such a difference may be related to the potential immune preservation in the W-SPDP group^18^. At least, the absence of a survival advantage in the DPS group may suggest that splenectomy does not confer any oncological advantage in the surgical management of left-sided PNET. Future studies should be focused on long-term survival.

Regarding surgical quality, there was no difference in terms of R0 resection rates and regarding the median number of retrieved lymph nodes between W-SPDP and DPS. Although lymph nodes metastases are related to adverse tumour features, the relevance of lymphadenectomy in PNET management remains disputed^19–23^. According to current guidelines, lymphadenectomy is routinely recommended for PNET >3 cm and/or G2 PNET^3^. In such a situation, W-SPDP could rather be considered as a safe alternative to DPS, especially for tumours located at the body of the pancreas, with no suspected lymph nodes on preoperative imaging.

Several points of limitation need to be addressed. The current study design and retrospective nature imply potential bias. For instance, besides missing histological data such as vascular invasion and perineural invasion, other data were lacking. Mostly, reasons driving the choice of each surgical technique would have been of interest. Indeed, such a choice might have been driven by surgeon preference, patient characteristics, or tumour features. As previously mentioned, given the limited duration of follow-up, long-term outcomes analysis should be carefully interpreted. Many left-sided PNET can also be removed using parenchyma-sparing resection such as enucleation or central pancreatectomy to reduce postoperative pancreatic insufficiency^24,25^. As such, the whole story of left-sided PNET management is not fully captured in the present cohort. K-SPDP is classically indicated for small PNET, when routine lymphadenectomy is not warranted^26^. In the current study, K-SPDP was mostly performed in patients with significantly smaller PNET and more frequently G1 as compared to W-SPDP and DPS patients. Conversely, Warshaw and DPS were performed in patients with more advanced tumour features, similar in terms of tumour size and grade between both techniques. Consequently, the current study focused on differences between W-SPDP and DPS. Further studies would be justified to determine the adequate SPDP technique for small PNET. Another key limitation of the study was the low number of retrieved lymph nodes which limited the analysis on the nodal disease. However, this number was quite similar to those reported in existing series for PNET or intraductal papillary mucinous neoplasm^17,27^.

In conclusion, SPDP could be routinely considered as an option for left-sided PNET. In situations warranting lymphadenectomy and in the absence of spleen involvement, W-SPDP could stand as a safe alternative to DPS. Like prospective studies in pancreatic ductal adenocarcinoma, the added value of splenectomy during DP for PNET would have to be defined based on oncological criteria such as the rate of splenic hilum lymph node metastasis and survival outcomes^28^.

## Collaborators

Mustapha Adham, Marie André, Philippe Bachellier, Louise Barbier, Thomas Bardol, Zineb Cherkaoui, Thibault Durin, David Fuks, Zaher Lakkis, Cloé Magallon, Fabien Robin, Edouard Roussel, Ecoline Tribillon, Stéphanie Truant, and Stylianos Tzedakis

## Supplementary Material

zraf038_Supplementary_Data

## Data Availability

Data can be made available upon reasonable request.
