# Supplementary material for: Benefit of splenectomy in distal pancreatectomy for neuroendocrine tumours: multicentre retrospective study
Source: BJS Open. 2025 May 13;9(3):zraf038. doi: 10.1093/bjsopen/zraf038 (PMC12070039; doi:10.1093/bjsopen/zraf038)
Supplement: zraf038_Supplementary_Data [file zraf038_supplementary_data.zip › zraf038_Supplementary_Data.docx]

Supplementary Figure 1. Flow chart
